# Role of Physical Bolus Properties as Sensory Inputs in the Trigger of Swallowing

**DOI:** 10.1371/journal.pone.0021167

**Published:** 2011-06-27

**Authors:** Marie-Agnès Peyron, Isabelle Gierczynski, Christoph Hartmann, Chrystel Loret, Dominique Dardevet, Nathalie Martin, Alain Woda

**Affiliations:** 1 Unité de Nutrition Humaine-UMR 1019, INRA, Université d'Auvergne, CRNH Auvergne, Clermont-Ferrand, France; 2 Nestlé Research Center, Vers-Chez-Les-Blancs, Lausanne, Switzerland; 3 Centre de Recherche en Odontologie Clinique – CROC, EA 3847, Université d'Auvergne, Clermont-Ferrand, France; 4 Service d'Odontologie, CHU, Clermont-Ferrand, France; The University of Western Ontario, Canada

## Abstract

**Background:**

Swallowing is triggered when a food bolus being prepared by mastication has reached a defined state. However, although this view is consensual and well supported, the physical properties of the swallowable bolus have been under-researched. We tested the hypothesis that measuring bolus physical changes during the masticatory sequence to deglutition would reveal the bolus properties potentially involved in swallowing initiation.

**Methods:**

Twenty normo-dentate young adults were instructed to chew portions of cereal and spit out the boluses at different times in the masticatory sequence. The mechanical properties of the collected boluses were measured by a texture profile analysis test currently used in food science. The median particle size of the boluses was evaluated by sieving. In a simultaneous sensory study, twenty-five other subjects expressed their perception of bolus texture dominating at any mastication time.

**Findings:**

Several physical changes appeared in the food bolus as it was formed during mastication: (1) in rheological terms, bolus hardness rapidly decreased as the masticatory sequence progressed, (2) by contrast, adhesiveness, springiness and cohesiveness regularly increased until the time of swallowing, (3) median particle size, indicating the bolus particle size distribution, decreased mostly during the first third of the masticatory sequence, (4) except for hardness, the rheological changes still appeared in the boluses collected just before swallowing, and (5) physical changes occurred, with sensory stickiness being described by the subjects as a dominant perception of the bolus at the end of mastication.

**Conclusions:**

Although these physical and sensory changes progressed in the course of mastication, those observed just before swallowing seem to be involved in swallowing initiation. They can be considered as strong candidates for sensory inputs from the bolus that are probably crucially involved in the triggering of swallowing, since they appeared in boluses prepared in various mastication strategies by different subjects.

## Introduction

The oral processing of food involves two functions: mastication and swallowing, both controlled by a specific central pattern generator (CPG) located in the brainstem [1], [2]. Extensive sensory information from the oral cavity is needed for their respective regulation and adaptation to bolus changes in the mouth, and safe swallowing obviously relies on these sensory inputs [1]–[4]. Numerous sensory inputs are produced when the food is introduced into the mouth, and they evolve during chewing when the food is progressively transformed into a bolus suitable for swallowing. Using this sensory information on the bolus state at any time in the chewing sequence, the CPGs can decide either to continue mastication for further food transformation, or to stop chewing, to control lingual forces and movements to propel the bolus to the pharynx [4]–[6]. Among the tactile stimuli that are major sources of information about the bolus state, the reduction of food has long been recognised as critical in producing the stimulus marking both the endpoint of mastication and the starting-point of swallowing. The particle size distribution in a ready-to-swallow bolus was first named the “swallow threshold” [7], [8]. Later, the role of lubrication due to both saliva and fluids from foods was considered as a further source of sensory information from the bolus [9]. For other authors using modelling [10], the optimum time for swallowing coincides with a peak in cohesive forces between food fragments. The role of bolus rheology has recently been highlighted for various foods in experiments relating food rheological behaviour to the ease of swallowing perceived by subjects [11], [12]. Nevertheless, the swallowing threshold concept has remained mainly theoretical, since no experiment has investigated the multiple physical dimensions of the ready-to-swallow bolus and their relation to the simultaneous physiological and sensory events. Although a broad range of boluses is acceptable for solid foods, especially with voluntary swallowing, it is clear that the bolus has to meet certain requirements to be swallowed [10], [13], [14].

This study was designed to analyse the physical properties of the bolus at various time points in the chewing process. Boluses collected in normo-dentate subjects from the start of mastication until swallowing were analysed for granulometry and rheological behaviour, and sensory textural perception was analysed along the masticatory sequence. Particle size distribution was measured by sieving and mechanical response was evaluated using a rheological method. This rheological test was a double compression test developed to simulate two successive bites applied on the food sample during mastication and is based on the classification of food mechanical properties described during sensory experience [15]. We hypothesized that the physical properties characterizing the bolus at the end of chewing formed the stimuli responsible, at least in part, for triggering swallowing.

## Methods

### Subjects

The study was approved by the French Ethics Committees (CPP-AU704, DGS-2007-0268). The subjects gave their written informed consent after receiving an explanation of the study goals. The sensory test was conducted under institutional Nestlé management approval. Twenty subjects (10 females, 10 males, age 23±2 years) were enrolled for analysis of physical properties of the bolus, and 25 others (13 females, 12 males, age 27±4 years) for the sensory analysis. All the subjects were students recruited through advertising and selected on strict dental criteria (healthy complete dentition, no masticatory disorders, normal occlusion, and no current or recent dental or orthodontic treatment).

### Food bolus collection

Portions of 3 g of petal wheat-flake cereals were prepared before each session and presented to the subject in a teaspoon. Each subject attended four sessions for the whole protocol over four weeks. Each session took place at least 1 h and no more than 1 h 30 min. after the most recent meal. The first session was used for training the subject to expectorate and for verifying that expectoration did not change the time of spontaneous swallowing under experimental conditions. For this purpose, the first three samples were chewed and swallowed. The next eight samples were chewed and expectorated at the time the subject felt the need to swallow. Number of cycles and duration of the masticatory sequence were used to verify that there was no change in the swallowing time. The second and third sessions were designed to collect boluses for two series of mechanical measurements performed in two different conditions, and the fourth to collect boluses for granulometric analysis. The subject sat comfortably, had water at will to drink between samples and was instructed to chew as usual. During these three sessions 12 samples were chewed. The first sample was chewed and naturally swallowed. The next two samples were chewed and expectorated. These three normal masticatory sequences were used for determining the characteristics of the complete masticatory sequence needed to reach swallowing naturally (*N*_swallow_). The number of cycles and the duration of the masticatory sequence were measured and the masticatory frequency was calculated. These physiological variables served as the individual time references of a complete masticatory sequence. The nine other samples were naturally chewed, but the masticatory sequences were stopped experimentally before the end of the sequence. The different time points for stopping the sequence were preset fractions of the *N*_swallow_ value (Table 1) and were assigned at random to subjects blind to their sequence order. For rheological measurements, the subject expectorated most of the bolus. For granulometric analysis, the particles remaining after expectoration were collected by rinsing with water (40 ml) and added to the expectorated bolus. All the boluses were analysed immediately after collection.

**Table 1:** Labelling of the different boluses collected along the masticatory sequence.

Bolus	Labelling
B1c	bolus collected after 1 masticatory cycle
B3c	bolus collected after 3 masticatory cycles
B1/4	bolus collected after 1/4 of the masticatory sequence (Nswallow x 1/4)
B1/3	bolus collected after 1/3 of the masticatory sequence (Nswallow x 1/3)
B1/2	bolus collected after 1/2 of the masticatory sequence (Nswallow x 1/2)
B5/8	bolus collected after 5/8 of the masticatory sequence (Nswallow x 5/8)
B6/8	bolus collected after 6/8 of the masticatory sequence (Nswallow x 6/8)
B7/8	bolus collected after 7/8 of the masticatory sequence (Nswallow x 7/8)
Bsw	bolus collected at the end of the complete masticatory sequence (Nswallow)

### Bolus mechanical analysis

**Figure 1:**
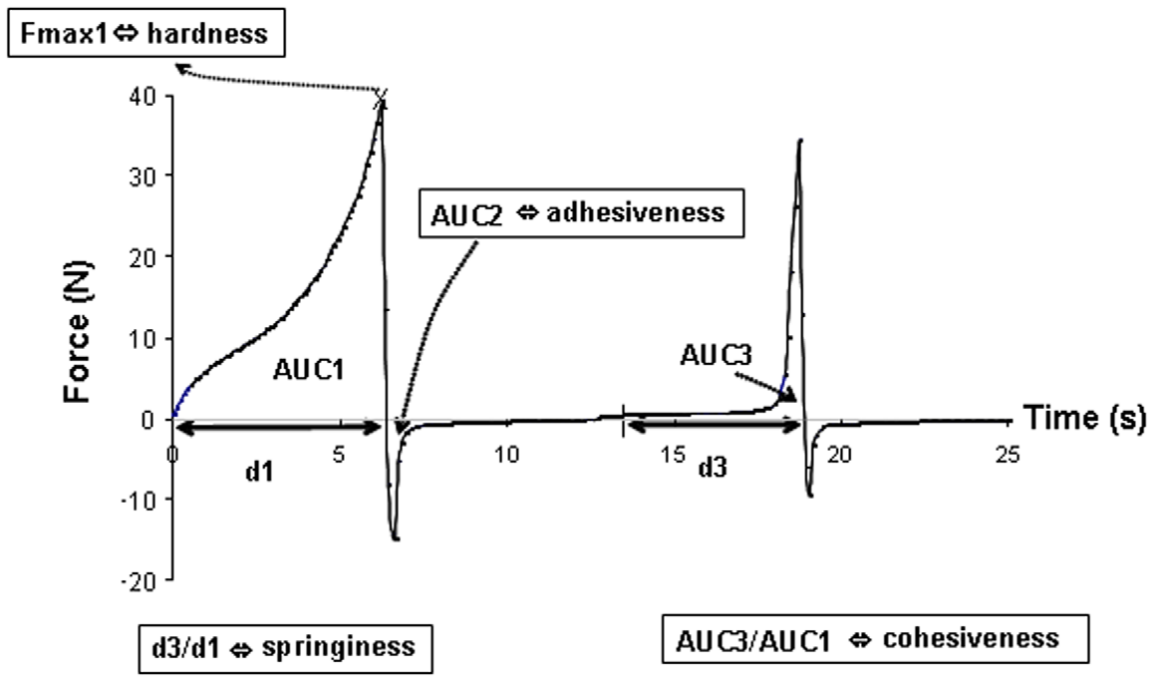
Example of a texture profile analysis (TPA) curve. This kind of curve was obtained for a cereal food bolus collected after mastication. Hardness is taken as the maximal force reached during the first compression. Adhesiveness is the area under the negative curve after the first compression, representing the work done to pull the food bolus apart in tension. Cohesiveness is the ratio of the area under the second compression curve to the area under the first compression. Springiness is the duration of the contact between the piston tool and the bolus during the second compression divided by the duration of the contact during the first compression.

Rheological properties of boluses were measured by the texture profile analysis (TPA) method using an Instron (mini55, UK) equipped with a flat piston head (ø 28 mm), a cylindrical cup (int. ø 35 mm) and a 500 N load cell. The bolus underwent two successive compression cycles performed at a constant displacement rate of 50 mm/min [16]. Compression ratios of 65% and 20% of deformation were chosen as these are classically used to test foods in destructurant and nondestructurant conditions. A given deformation condition was applied on boluses collected during the same session. These two conditions allowed full bolus characterization. Hardness, cohesiveness, springiness and adhesiveness values were chosen as bolus mechanical characteristics because they are the usual physical properties extracted from the force-time curve obtained with the TPA test (Figure 1). In addition, the TPA test has been developed and validated to analyse such mechanical characteristics in regard to sensory experimentation [15], [16].

### Bolus granulometric analysis

Particle size distribution in boluses was determined by dry manual sieving. The bolus was poured onto a 0.3 mm nylon cloth (Sefar-Nitex, Switzerland), washed in running water to eliminate saliva, and left for 2 h at 30°C in a ventilated incubator. The dried bolus was poured onto a stack of 7 sieves with apertures of 4, 2.5, 2, 1.4, 1, 0.8 and 0.4 mm (Saulas, France) and manually sieved using a paintbrush. The particles retained on each sieve were weighed and results expressed as a cumulative curve using the particle mass falling through each sieve. From each curve, the median particle size *d*_50_, defined as the aperture of a theoretical sieve through which 50% of the mass could pass, was determined.

### Bolus sensory analysis

Subjects from the sensory panel were instructed to evaluate several texture attributes (brittleness, crispness, crackliness, dryness, grittiness, hardness, lightness, stickiness) on the same cereals (3 g portions). These attributes were determined by the sensory panel as the most representative of their sensory experiences. These attributes are those always used for sensory description of cereals. This was done using the method of temporal dominance of sensation [17], which identifies the dominant perception at each time during the eating period. The panellists were asked to eat the portion and indicate the attribute they perceived as dominant among the eight texture attributes at any time during the mastication. An attribute was considered as dominant until another one was indicated by the subject. Each subject followed the procedure twice, *i.e.* on two portions of cereals in order to increase the number of observations and consequently the power of the test. This procedure produced 50 observations. Data was acquired on a computer with Fizz software (Biosystèmes, 1990). The number of responses expressing each dominant perception was calculated at several time points in the complete sequence. The same time points were chosen for sensory analysis and granulometric and rheological measurements (Table 1).

### Data analysis

Mechanical and granulometric data were expressed as absolute values at each preset time point of the masticatory sequence. Sensory data were expressed as dominance rate, *i.e.* the proportion of responses eliciting the same attribute as dominant at the same time points.

Statistical analyses were performed using SPSS (v11.5). Normality of the distributions of dependent variables was verified. As a prerequisite for subsequent analysis, reproducibility of masticatory variables (number of cycles at swallowing, duration of sequence and masticatory frequency) was verified by a concordance test (intraclass correlation) between sessions (ICC value for number of cycles  = 0.9709, P<0.001), between complete sequences ending with swallowing or complete sequences ending with bolus expectoration (ICC value for number of cycles  = 0.9812, P<0.001; ICC value for frequency  = 0.9596, P<0.001) and between normal and experimentally stopped sequences (ICC value for frequency  = 0.9361, P<0.001). For this later analysis, values for B1c and B3c boluses were not included, since the sequences were too short for exact measurements. Differences between subjects were tested by a two-way ANOVA (session, subject) using a general linear model (GLM).

Five two-way ANOVAs in mixed models (bolus as fixed effect, subject as random effect) were performed to test for rheological and granulometric differences (hardness, cohesiveness, springiness, adhesiveness, *d*_50_ *)* between boluses collected at different time points during mastication. When significant differences were observed (*P*<0.05), mean values for two consecutive bolus were compared using the Student-Newman-Keuls test.

For the 50 observations obtained from the sensory experience, the dominance perception was expressed for each texture attribute as a percentage of observations. A two-tailed test based on the normal approximation was applied to determine whether the number of reports from subjects perceiving a given attribute significantly differed between two consecutive bolus as the sequence progressed [18]. An α-risk of 0.15 was chosen to express significances: subjects had to choose among eight attributes at each time and a high level for α-risk pinpointed noteworthy trends.

Data were expressed in absolute values during the masticatory sequence. Slopes between data from two consecutive boluses were then used to find the time at which notable changes in bolus properties appeared during the masticatory sequence.

## Results

### Masticatory sequences

Swallowing of a cereal bolus was carried out after 39±12 masticatory cycles performed in 26±7 s with a frequency of 1.5±0.15 s^−1^. The well-known broad between-subject variability in chewing was confirmed (*P*<0.001) for these variables. The masticatory frequency was not different between complete and experimentally interrupted sequences. Masticatory sequences ending with expectoration were not different from those ending with normal swallowing.

### Granulometric and rheological bolus characteristics

**Figure 2:**
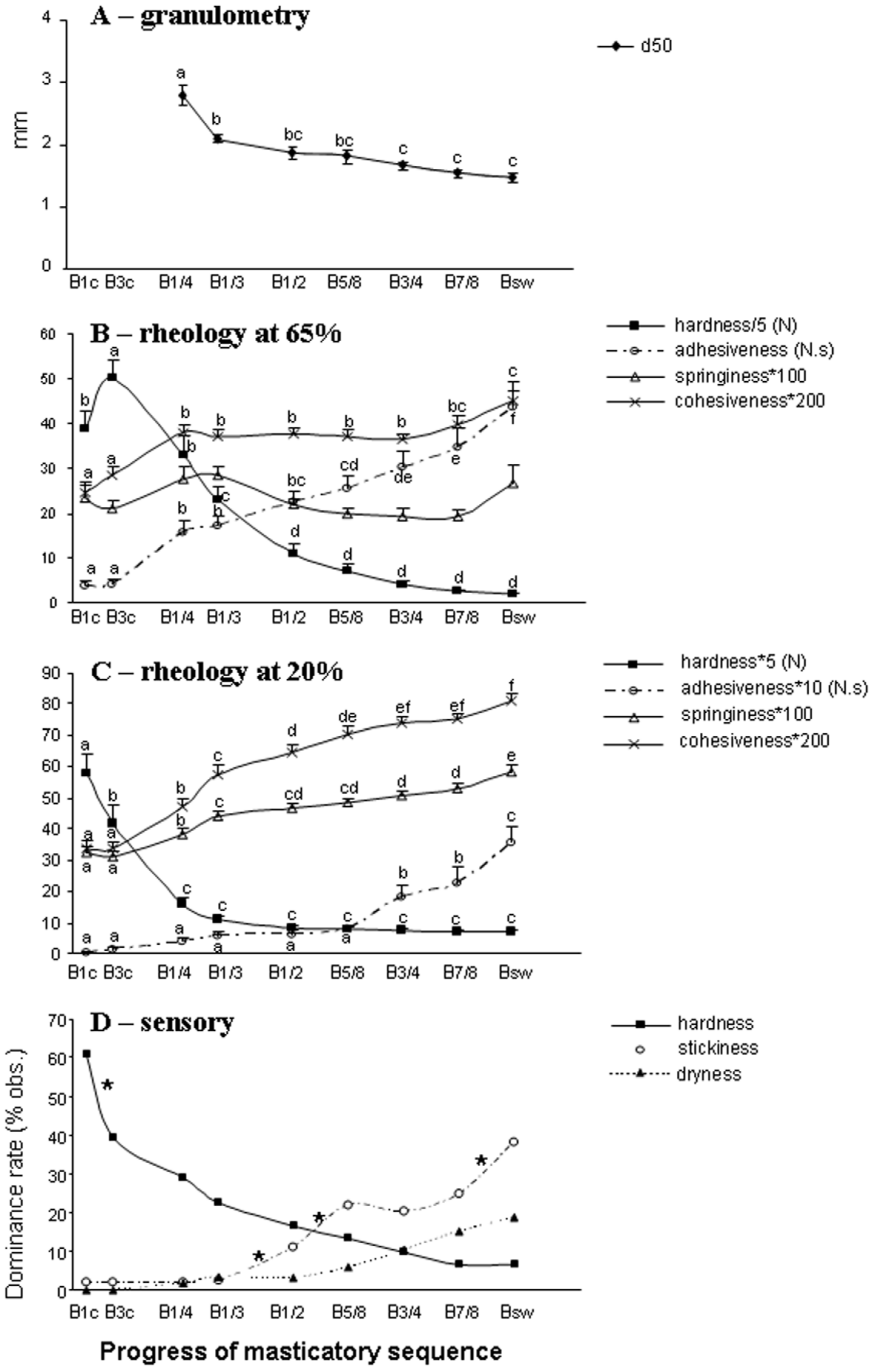
Bolus characteristics analysis. Median particle size (*d*_50_ *)* [A], hardness (in N), adhesiveness (in N.s), springiness and cohesiveness measured at 65% [B] or 20% [C] of deformation of the bolus are presented for each bolus collected at several time points between the beginning and end of the complete masticatory sequence (B1c to Bsw). Dominance rate for hardness, stickiness and dryness perceptions were calculated from 50 observations and are shown at the same time points as physical measurements [D]. Springiness and cohesiveness are dimensionless. Significant differences between two consecutive boluses are shown with lower-case letters for granulometric and rheological data (*P*<0.05), and with * for sensory data (*P*<0.15). Although a very small significance was observed for springiness measured at 65% deformation, no significant difference was noted with SNK test between consecutive boluses. Results for physical measurements are means ± SEM (*N* = 20). Points obtained for a given variable are joined up to improve readability.

**Figure 3:**
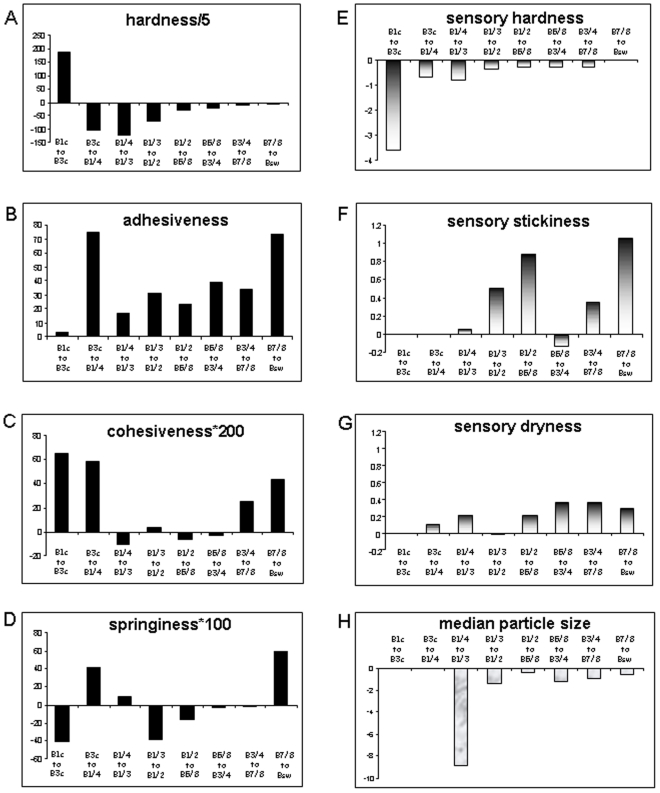
Sequential analysis of bolus characteristics. Changes observed during the progress of masticatory sequence in mechanical hardness (A), adhesiveness (B), cohesiveness (C) and springiness (D) calculated on data obtained from TPA performed at 65% deformation, associated changes in proportions of subjects perceiving hardness (E) stickiness (F) and dryness (G) as being dominant in the bolus, and bolus median particle size (H). Bars represent slopes calculated between values from two consecutives boluses.

The *d*_50_ value fell sharply in the first half of the masticatory sequence and then more slowly, down to a mean value of 1.52 mm for the swallowable bolus (*P*<0.001; Figures 2A, 3H). Hardness significantly decreased from the beginning of the sequence until swallowing (*P*<0.001; Figures 2B–C, 3A). The largest decrease occurred during the first third of the sequence. Conversely, adhesiveness, cohesiveness and springiness significantly increased as the masticatory sequence proceeded until swallowing at both 20% and 65% deformation (*P*<0.001). Changes in adhesiveness, cohesiveness and springiness were still significant between the last two boluses, except for springiness measured at 65% and cohesiveness at 20% (Figures 2B–C and 3B–C–D).

### Sensory characteristics of the bolus

Among the cereal texture attributes perceived by the subjects during the masticatory sequence, the dominance rate calculated for hardness and stickiness displayed the same qualitative time course as the mechanical characteristics assumed to be associated with it, *i.e*. hardness and adhesiveness measured with TPA (Figure 2D). Hardness was perceived as dominant at the beginning of the sequence (Figure 3E). Stickiness was perceived as dominant as the mastication proceeded towards swallowing, with an optimum between B7/8 and Bsw (P<0.15; Figures 2D, 3F). Although non-significant, dryness increased slightly in the second part of the sequence (Figure 3G). None of the other texture attributes were perceived as dominant during the second part of the sequence.

## Discussion

Swallowing has rarely been studied to determine what properties a bolus must display to be propelled towards the oesophagus. Most studies have focused either on effects of viscosity on flow through oral and pharyngeal compartments [5], [19]–[22] or on effects of bolus volume on propulsion characteristics [19], [23]. It is generally assumed that to be safely transferred into the oesophagus, a bolus needs suitable rheological and surface properties as well as particle size [24]–[28]. The physical and gustative properties of the bolus serve as stimuli and initiate sensory messages to the masticatory and swallowing central pattern generators (CPGs). The perception of changes in bolus characteristics [29]–[32] as the sequence proceeds is thought to be read by these CPGs and could finally indicate a ready-to-swallow bolus at the end of mastication. These changes and their perception have been conceptualized as the “swallow threshold” [7] but the physical nature of the stimuli used has never been quantitatively determined. Only very limited approaches have been taken [11], [12]. Quantitative data have been obtained in this study for the first time, albeit in an indirect way, and these bolus characteristics can be selected for further investigation. It must be noted, however, that the measures were performed at the end of the processing phase and before stage II transport of swallowing [33]. It can be assumed that the bolus properties do not undergo extensive physical changes during the stage II transport. Even so, there is a possibility that intentional holding of the food bolus in the mouth, overriding stage II transport, modified the timing of the bolus transformation [34]. This study suggests that some physical changes are still occurring in the bolus at the time it can be swallowed and that at least some of these changes are perceived.

The changes in springiness, adhesiveness and cohesiveness of the cereal bolus during the masticatory sequence were progressive but more pronounced between the last two boluses. Thus a modification of the bolus still occurred just before swallowing. This enriches the traditional swallowing-threshold concept, emphasizing the importance of rheological properties such as cohesiveness and adhesiveness for a safely swallowable bolus.

Sensory attributes and physical properties are two different dimensions and it is not surprising that some of them are not described by the same term. However, the TPA test simulates two bites on the food to reflect the chronological order of appearance of sensory manifestations of textural properties. It has therefore been developed and validated to analyse mechanical characteristics in regard to sensory experiments and it is currently assumed that these refer to correspondences between mechanical characteristics and some sensory descriptors [15], [16]. Cohesiveness can be defined as the resulting forces inducing particles to stick together and constitute the bolus as an entity [35]. Cohesiveness is probably reflected in perceived stickiness [36]. Adhesiveness can be described as resulting from external forces due to attraction between the bolus and mouth parts. It probably depends on food properties and saliva characteristics. Stickiness can be taken as the sensory experience of mechanical adhesiveness. The tendency for an increased dryness perception at the end of the masticatory sequence could be linked to exchange between the solid and aqueous phases in the bolus. During mastication, progressive saliva absorption in the bolus draws liquid, probably increasing dryness perception at the end of the sequence. This perception may also participate in swallowing initiation. The progressive adding of saliva could also lend the bolus non-Newtonian behaviour that may make it easier to swallow [11].

This study shows that particle size and hardness are not the only decisive factors in the swallowing threshold, since *d*_50_ and hardness values changed little from the middle of the masticatory sequence. Particle size [8], [37], lubrication by saliva and bolus wetting [38] are initial contributing factors by which the final rheological values of swallowing threshold can be obtained. The several thresholds critical for swallowing may not be reached simultaneously in a bolus; swallowing threshold is probably an integrative process combining the perception of the various bolus properties enabling swallowing. Obviously the swallowing initiation mechanism also encompasses proprioceptive information on dynamic activity from muscles or receptors and other sensory experiences such as gustative or acoustic cues [6]. It has thus been shown that muscular activity at the end of the masticatory sequence is the same for several boluses produced for food samples differing only in initial hardness [39]. Proprioception from muscles is informative about bolus hardness and is probably associated with a swallowing decision.

Evidently, the swallowing threshold comprises many components. As the formation of the swallowable bolus is assumed to be a key driving constraint to avoid dangerous aspiration, each individual uses their physiological means to chew a given food until a safe bolus is made and the swallowing threshold is reached. This “driving constraint” concept is illustrated by three converging studies performed with ten different healthy subjects. They highlighted a narrow variability in particle size of the swallowable food bolus [28], [37], [40] in contrast to a broader variability of the physiological parameters. However, a greater variability of the particle sizes in the swallowable bolus has been reported in studies with more subjects [7], [41], indicating that other determinants, *e.g.* rheological/saliva content, are probably involved in the swallowing threshold concept.

Swallowing dysfunction is a prominent problem in several populations and a cause of morbidity/mortality [42]. Persons with impaired mastication such as edentate subjects or persons with Down syndrome have been shown to produce pre-swallow boluses containing many large particles [43], [44]. Consequently, their bolus properties cannot reach the swallowing threshold levels, leading to dietary changes [45], [46] or to the swallowing of an ill-formed bolus [8], [43], [44]. Hence knowing the bolus characteristics needed for safe swallowing should be a strong research purpose [47], [48], [49]. A new indicator (MNI) has been established to differentiate subjects with normal and impaired mastication [44]. Further studies are still needed to analyse swallowing competence according to both the physical nature of ingested food as used sensory inputs and individual ability to form a swallow-safe bolus.
